# A realist evaluation of the role of communities of practice in changing healthcare practice

**DOI:** 10.1186/1748-5908-6-49

**Published:** 2011-05-23

**Authors:** Geetha Ranmuthugala, Frances C Cunningham, Jennifer J Plumb, Janet Long, Andrew Georgiou, Johanna I Westbrook, Jeffrey Braithwaite

**Affiliations:** 1 Centre for Clinical Governance Research, Australian Institute of Health Innovation, University of New South Wales, Sydney, NSW 2052, Australia; 2 Centre for Health Systems and Safety Research, Australian Institute of Health Innovation, University of New South Wales, Sydney, NSW 2052, Australia

## Abstract

**Background:**

Healthcare organisations seeking to manage knowledge and improve organisational performance are increasingly investing in communities of practice (CoPs). Such investments are being made in the absence of empirical evidence demonstrating the impact of CoPs in improving the delivery of healthcare. A realist evaluation is proposed to address this knowledge gap. Underpinned by the principle that outcomes are determined by the context in which an intervention is implemented, a realist evaluation is well suited to understand the role of CoPs in improving healthcare practice. By applying a realist approach, this study will explore the following questions: What outcomes do CoPs achieve in healthcare? Do these outcomes translate into improved practice in healthcare? What are the contexts and mechanisms by which CoPs improve healthcare?

**Methods:**

The realist evaluation will be conducted by developing, testing, and refining theories on how, why, and when CoPs improve healthcare practice. When collecting data, context will be defined as the setting in which the CoP operates; mechanisms will be the factors and resources that the community offers to influence a change in behaviour or action; and outcomes will be defined as a change in behaviour or work practice that occurs as a result of accessing resources provided by the CoP.

**Discussion:**

Realist evaluation is being used increasingly to study social interventions where context plays an important role in determining outcomes. This study further enhances the value of realist evaluations by incorporating a social network analysis component to quantify the structural context associated with CoPs. By identifying key mechanisms and contexts that optimise the effectiveness of CoPs, this study will contribute to creating a framework that will guide future establishment and evaluation of CoPs in healthcare.

## Background

With a focus on knowledge sharing and learning, communities of practice (CoPs) are being promoted in the healthcare sector as a means of improving practice and patient care [1]. By definition, a CoP is a group of people 'who share a concern, a set of problems, or a passion about a topic, and who deepen their knowledge and expertise on this area by interacting on an ongoing basis' [2]. It is argued that CoPs nurture and harness knowledge, particularly in terms of promoting the exchange of tacit knowledge, and drive innovation to help individuals and organisations improve practice and performance [3-5]. Such claims have contributed to the widespread adoption of CoPs in healthcare and other sectors seeking to effectively manage knowledge in order to improve organisational performance.

Reflecting the increased uptake of CoPs in the healthcare sector, the number of peer-reviewed papers reporting on CoPs is steadily increasing [6], and includes the publication of two systematic reviews in 2009 [7,8]. Despite this increase, there is a lack of empirical evidence demonstrating the impact of CoPs in improving healthcare practice. Much of the published literature is limited to describing the establishment or activities of CoPs [6,9]. If organisations and sponsors are to foster CoPs for their value in knowledge management and for improving organisational performance, there is a need to understand better the role of CoPs in improving healthcare practice. To this end, a realist evaluation of CoPs is proposed.

In contrast to traditional evaluation methods that examine the success of an intervention based on whether or not a predefined outcome has been achieved, the realist approach seeks to answer the questions -- how, why, and when does the intervention work [10]? A realist evaluation is a theory-driven approach to understanding what it is about a program that achieves a particular outcome in one setting and a different outcome in another. It is well suited for social interventions where outcomes are determined through stakeholder action and interaction, which in turn is likely to be influenced by social and cultural norms [10,11]. Underpinned by the principle that context (C) will trigger mechanisms (M) to yield outcomes (O), a realist evaluation goes beyond focussing purely on inputs and outputs; it involves exploring and identifying the mechanisms by which the inputs are converted inside a 'black box' into outputs, and recognises the need for particular conditions (or contexts) to be present inside the black box for the causal mechanisms to be triggered and yield a particular outcome. The relationship between context, mechanism, and outcome is presented as a 'CMO configuration' [10]. Based on these principles, the objective of this study is to identify CMO configurations that will explain the role of CoPs in improving healthcare practice. This objective will be achieved by seeking to answer the following questions: What outcomes do CoPs achieve in the healthcare sector? Do these outcomes translate into improved practice in healthcare? What are the contexts and mechanisms by which CoPs impact on improved practice in healthcare?

## Methods

This realist evaluation will be conducted in four stages as shown in Table 1 corresponding to the four components of the realist evaluation cycle (theory, hypotheses generation, observations, and program specifications) as described by Pawson and Tilley [10]. The stages will be undertaken sequentially such that the findings from each stage will inform the next stage, and the final stage will involve reviewing the findings from stage three to confirm, modify, or reject the theory-based hypotheses generated in stage two [12].

**Table 1:** Four-stage approach to the realist evaluation of communities of practice

Stage	Activities	Analysis	Purpose
1. Theory	• Systematic review of the literature • Semi-structured interviews with sponsors and facilitators of CoPs • Formulate CMO configurations	• Qualitative -- Identify themes and categorise as outcomes, mechanisms, and contextual factors • Formulate potential CMO configurations	Provide the theoretical basis for the realist evaluation
2. Hypotheses generation	• Generate hypotheses based on CMO configurations	Rephrase CMO configurations into hypotheses	Formulate hypotheses to be tested during stage three
3. Observation	• Online survey of CoP members	Quantitative -- • Identify CMO configurations that occur with regularity • Social Network Analysis	Test and accept, reject, or modify hypotheses Examine structure of professional and social relationships and flow of information and knowledge within the CoP
4. Program specification	• Review analysis from stage three	Refine theorised CMO configurations based on testing of hypotheses	Specify CMO configurations that explain how, when and why CoPs improve healthcare practice

An opportunistic sample of four CoPs will be identified, and sponsors, facilitators, and members of each CoP will be interviewed and surveyed using semi-structured interviews and online surveys. These four CoPs will act as case studies to enable the in-depth exploration required to understand the happenings within the 'black box' linking CoPs to improved practice.

### Stage one: theory

The first stage of the realist evaluation involves developing candidate theories on the role of CoPs in improving healthcare practice and potential CMO configurations. This begins with a systematic search and review of the healthcare literature identifying characteristics of CoPs and outcomes achieved. By focusing the review specifically on the healthcare sector, the information collected will be context specific. Our systematic review [6] identified particular features by which CoPs in the healthcare sector differ (Table 2), providing us with a starting point for formulating contexts and potential mechanisms by which CoPs in the healthcare sector influence change in practice.

**Table 2:** Characteristics of communities of practice identified from the literature [6]

Characteristic	Findings from the literature review
Membership and practice	• One becomes a member through shared practice [32] • CoPs help establish professional identity [7] • Members have a common goal or purpose [32] • Membership often crossed geographical, professional, and/or organisational boundaries [6] • Membership group and size is not fixed and can vary from time to time [6,33] • The focus of the group may vary over time [33]
Activities and communication methods	• Members exchange knowledge through formal and informal processes. Formal methods of interaction include face-to-face meetings within or external to usual workplace and/or virtual methods that include communication via email and/or blogs [6] • Social interaction, in person or through the use of communication technology, is an important feature of a CoP identity [7]
Origin	• Spontaneous origin or established as a management initiative [6] • CoPs have five stages of development: potential, coalescing, maturing, stewardship, and transformation [2]
Determinants of success	• A committed facilitator [6] • Shared purpose [34] • Commitment and enthusiasm from the members [34] • Endorsement of the CoP from senior management and alignment of the CoP objectives with the organisation goals [35,36] • A CoP with self-selected membership may be more successful than a CoP with externally appointed members [34] • Regular communication with, and interaction between members [37] • Developing relationships through face-to-face interactions, even to start with, is important [36] • Infrastructure to support the work of the CoP in terms of ease of access to knowledge or evidence [34]

The next activity in stage one is to interview sponsors and facilitators with the objective of identifying CMOs that will, in turn, be used to generate candidate theories on CMO configurations explaining the role of CoPs in improving healthcare practice. Additional data will be collected on resources offered to members and the means by which impact is assessed. The questions used to guide these interviews are in Additional File 1.

When collecting data from published literature and from interviews, contexts will be defined as the settings in which the CoP operates. To a large extent, these will be the characteristics of the CoP in terms of constitution of membership, level of maturity of the CoP, and activities organised by the CoP. Contexts will be also be determined by examining the connections, interactions, and knowledge flow that occur within each CoP. Mechanisms will be defined as the factors and resources that the CoP offers its members to influence a change in behaviour or action [12,13]. A mechanism may be an enabler or a disabler depending on the context.

To date, there is no consistency in the way in which outcomes of CoPs are defined or measured [6]. For the purpose of this study, an outcome will be defined as a change in behaviour or work practice that occurred, influenced by participating in a CoP activity or through accessing resources provided by the CoP. The change may be to a process (such as adoption of a new system or process, or reduced time to achieve a goal that is related to improved care); an innovation (such as development of a new product or technology that will improve the delivery of healthcare); or change in level of customer (patient) satisfaction [14]. Financial outcomes will not be considered due to the focus of this study on clinical practice. Individual as well as organisational level outcomes will be sought, recognising that improved organisational performance is achieved through changing the work practice of individuals who contribute to the organisation.

**Figure 1:**
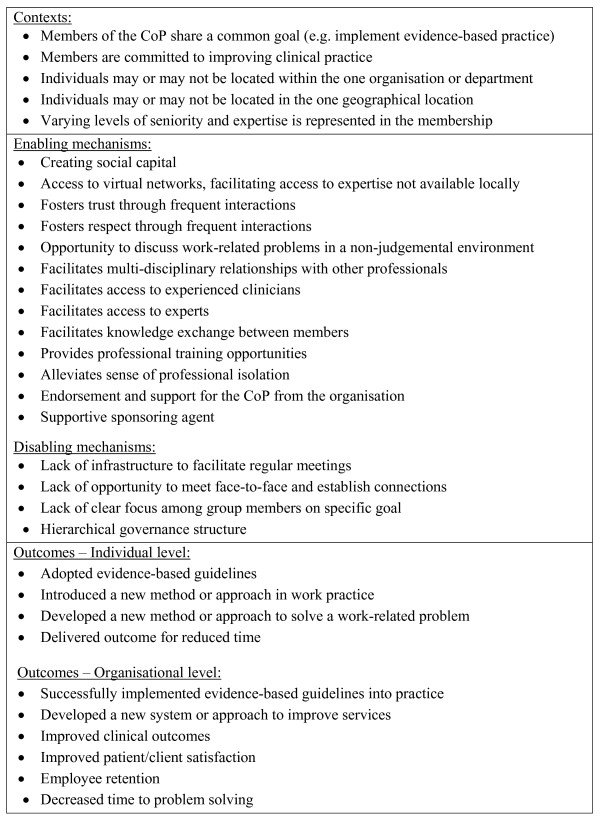
**Preliminary list of CMOs that potentially play a role in CoPs improving healthcare practice**.

The end product of stage one will be a list of CMOs and possible CMO configurations that explain the role of CoPs in improving healthcare. Figure 1 presents a preliminary list based on background research.

### Stage two: Hypotheses generation

The second (hypotheses generating) stage involves rephrasing the CMO configurations theorised in stage one as hypotheses for testing in stage three. These hypotheses will be framed around the theoretical relationships between specific CMOs that could explain the different outcomes of CoPs, depending on the context in which the CoP operates.

### Stage three: Observation

The hypotheses developed in stage two will be tested during stage three. All members of the participating CoPs will be asked to respond to a survey that will seek their level of agreement (using a five-point Likert scale) with hypotheses (see Additional File 2). Testing these hypotheses will help identify CMO configurations that occur with regularity, and provide possible explanations for the role of CoPs in improving healthcare practice.

The second part of the third stage will involve obtaining contextual information on the connections, relationships, and knowledge exchange that occur within a CoP. These are, by definition, essential elements of a CoP and form the context in which the hypotheses are tested. As identified in the literature and presented in Table 2 regular communication, interaction, and knowledge exchange between members are characteristics associated with CoPs. These elements have been linked to improved organisational performance through the concept of social capital as follows: Social capital is created by developing connections among practitioners that foster 'relationships that build a sense of trust and mutual obligation, and (by) creating a common language and contexts that can be shared by community members' [4]. The connections, relationships, and common contexts that generate social capital, in turn, positively impact on organisational performance [4]. The study will utilise social network analysis (SNA) methods to examine the professional connections and relationships within the CoP, represented by the strength of ties, so as to understand how the CoP network features may relate to improved healthcare practice. The social interactions that occur within the CoP will also be examined, recognising their role in the exchange of tacit knowledge [15].

Questions used to collect data on professional and social connections and knowledge exchange will be based on social network questions used by other researchers [16-18], modified where necessary to achieve the objectives of this study (See Additional File 3). The questionnaire will be validated by testing on a convenience sample of ten people with clinical practice and health management roles similar to those of members of the CoPs. Reliability will be tested using Kappa coefficient methods on test and retest of the instrument five days apart.

The network data collected in the study will be analysed using UCInet [19]. The NetDraw feature of this software allows visual examination of each of the relationships (*i.e*., professional connections, social interactions, and information and knowledge flow) for strength of connectedness based on the frequency of contact. It also aids the identification of cliques (or subgroups), cut-points (referring to a person whose departure will result in a break in flow of information/knowledge), and isolated individuals [20].

When surveying CoP members, knowledge will be defined as 'internali(s)ed or understood information that can be used to make decisions' [21]. Knowledge will be differentiated from information by the fact that '(k)nowledge is information possessed in the mind of individuals: it is personali(s)ed information (which may or may not be new, unique, useful, or accurate) related to facts, procedures, concepts, interpretations, ideas, observations, and judgements' [22]. The reason for differentiating knowledge from information is to examine how much of the information that CoPs provide their members is translated into knowledge that influences change in their own work and practice.

The analysis functions in UCInet will be used to quantify the connectivity and stability of the community by measuring degree, closeness and betweenness centrality, reciprocity of relationships, and multiplexity [23]. Degree centrality is the number of persons (or nodes) to which a particular person is directly linked; a higher score indicates a well connected person [24]. This measure will help identify key persons in the community, with the facilitator likely to score highly. A high average density score at the CoP level indicates a high level of direct links or interactions between members of the CoP. Closeness centrality recognises the importance of indirect connections for exchange of resources (such as knowledge) and measures the shortest path connecting a key node (CoP member, in this case) to any other node [25]. Betweenness centrality also takes into account the importance of indirect links in maintaining links between nodes not otherwise connected [25]. This, too, is relevant in terms of examining the flow of resources (such as information or knowledge) [24]. A CoP scoring highly in the knowledge exchange relationship would indicate high connectivity with little threat to knowledge exchange due to lost links. Reciprocity of each relationship will also be examined to identify bidirectional links, with suggestions that high level of reciprocity is characteristic of a more stable network [26]. The knowledge relationship will be examined further for path length, to assess the efficiency of information and knowledge flow and exchange within the CoP and will help identify how best to optimise this process [27].

As this study examines multiple relationships (that is, professional connections, social interactions, and information and knowledge flow), multiplexity will be examined as an indication of the strength of the link between members; with members linked by more than one relationship said to have stronger ties than those linked by one relationship [24,28,29].

### Stage four: Program specification

The fourth and final stage is program specification, during which the theorised role of CoPs in improving healthcare and potential CMO configurations from stage one will be reviewed in light of the findings in stage three. The CMO configurations that were supported with regularity will form the basis for specifying possible explanations for the role of CoPs in improving healthcare practice.

## Discussion

This paper describes a protocol that uses mixed methods to examine systematically and understand how, why, and when CoPs improve healthcare practice. Realist evaluation is being used increasingly in the healthcare sector, recognising the fact that programs and interventions requiring behavioural change operate within a complex social and cultural context, and that the operating context plays an important role in determining impact. In such circumstances, the traditional approach of evaluating success based on whether or not a pre-defined outcome is achieved does not provide decision makers with sufficient information to assess the value of the program outside the context in which it was tested. There is a need for methods that are able to tease out the mechanisms by which a program results in change, and study the interactions between these causal mechanisms and context [10,30].

Following the application of a realist approach to evaluate a modernisation initiative in the UK, Greenhalgh *et al*. discussed the difficulties in identifying the mechanisms of change and drawing realist conclusions around CMO configurations. They refer to this process as typically requiring 'a three-hour face-to-face meeting as well as lengthy email exchanges and numerous iterations and counteriterations' [31]. Work undertaken to date on this project affirms the difficulty of identifying mechanisms and outcomes, and generating the list presented in this paper has required lengthy discussions and iterations. Since our proposal also includes using SNA methods to examine the connections and knowledge exchanges within the CoP as a means of providing contextual information, we need to strike a fine balance between making significant demands on participants' time and securing the high response rates required for SNA. As a means of achieving this balance, we have chosen to limit the in-depth interviews and discussions to facilitators and sponsors of CoPs. Members will participate in testing the hypotheses generated by the discussions and will respond to the SNA questions. To help this process further and taking into consideration the length of the hypotheses testing survey, the SNA survey will be administered at a later date.

A challenging aspect of developing this study protocol was identifying and defining an outcome that would demonstrate the impact of CoPs in improving work practice. A finding from our systematic review [6] was that the vast majority of existing research had assessed impact though self-reported perceived benefits, with very limited effort to substantiate these claims through triangulation. This study will attempt to overcome this limitation by defining an outcome as a demonstrated change in work practice at the individual member level as well as the organisation level. The difficulty in drawing conclusions around CMO configurations will be addressed to some extent by looking for patterns that occur with regularity supporting the occurrence of such causal interactions.

The realist evaluation method, by seeking to understand how, why, and when a program works, is well suited for separating out and examining the multiple components in a program individually and in the context of the program. This feature is particularly useful given the difficulty experienced in directly attributing outcome to a CoP in studies that have measured and reported outcomes from multi-faceted interventions [6]. CoPs offer more than one resource to their members with the intention of facilitating knowledge creation and sharing. Knowing the role that each of these components play in influencing change in healthcare practice will help maximise value and return on investment in CoPs.

This study further enhances the value of realist evaluations by incorporating a SNA component to quantify the structural context associated with CoPs. To our knowledge, these two methods have not been previously combined. By examining the connections and relationships that occur within the community or network, SNA methods quantify the structural component of the context within which CoPs operate.

Overall, this paper proposes a research study to understand the complexity of CoPs, taking into consideration its multi-component nature and the influence of context in determining impact. The systematic approach proposed will help identify key mechanisms that operate within particular contexts, which in turn will help optimise the establishment and effectiveness of CoPs. The study will contribute to creating a framework that will guide the future development and evaluation of CoPs in the healthcare sector [9].

## Competing interests

The authors declare that they have no competing interests.

## Authors' contributions

JB and JIW conceptualised the overarching research project and are the chief investigators of the research grant funding this research activity. GR developed the study protocol presented in this paper in consultation will all other co-authors and wrote the first draft. All authors provided input into various aspects of the study, provided ongoing critique and approved the final version of the manuscript.

## Supplementary Material

Additional file 1 **Questions guiding interviews with sponsors and facilitators - for stage 1 of the realistic evaluation**. Click here for file

Additional file 2 **Survey of CoP members to test context, mechanism and outcome configurations**. Click here for file

Additional file 3 **Survey of CoP members to map the current structure, available expertise and knowledge exchange**. Click here for file
