# Supplementary material for: A realist evaluation of the role of communities of practice in changing healthcare practice
Source: Implement Sci. 2011 May 23;6:49. doi: 10.1186/1748-5908-6-49 (PMC3120719; doi:10.1186/1748-5908-6-49)
Supplement: Additional file 1 — Questions guiding interviews with sponsors and facilitators - for stage 1 of the realistic evaluation. [file 1748-5908-6-49-S1.PDF]

Additional File 1: Questions guiding interviews with sponsors and facilitators - for stage 1 of the realistic evaluation

(Interviews will be conducted face-to-face or by telephone)

Sponsor interview

The purpose of this interview is to understand the role that communities of practice (CoP) have in improving the delivery of healthcare. We are trying to identify outcomes achieved by CoPs in the healthcare sector, and understand some of the mechanisms by which these outcomes are achieved.

Outside of the phrase 'communities of practice', the term 'practice' is used such that it refers to the carrying out of work as it relates to one's professional role.

1. In your opinion, do communities of practice have a role in improving work practice in health care?
2. What is the role of the sponsoring agent?
3. From a sponsor perspective, what was the purpose of establishing this CoP?
4. In your opinion, what is it about the CoP that brings about a change in work practice?
5. What resources do you think it offers its members to influence change in behaviour and outcome?
6. In your opinion, how do you think CoPs facilitate the exchange of knowledge and best practice?
7. From a sponsor's perspective, how do you see the CoP benefiting its members as individuals?
8. Can you give two examples of such benefit to individual members?
9. From a sponsor's perspective, how do you see the CoP benefiting the organisations for which the members work?
10. Can you give two examples of such benefit at an organisational level?
11. Again from a sponsor's perspective, do you see the CoP having any national significance? If so, how?
12. In your opinion, what has been the single most major achievement of this CoP?
13. From a sponsor's perspective, what are the main barriers to the CoP facilitating the process of improving practice in health care?
14. From a sponsor's perspective, what are some factors that facilitate the role of the CoP in improving practice in healthcare?
15. Would you like to make any comments about the role of CoPs in improving organisational performance in healthcare?

Facilitator interview

The purpose of this interview is to ascertain the characteristics, goals and outcomes of the community of practice (CoP) that you facilitate, and to explore causal mechanisms that may be triggered by the contexts in which the CoP operates.

1. In which year was the CoP established?
2. What is the history behind the CoP being established?
3. What was the goal of the CoP when it was first established?
4. Has the goal of the CoP changed since its inception?
5. Who decides on the goal?
6. How many members did the CoP start with?
7. How many members does the CoP have at present?
8. How does one become a member of the CoP?
   - ☐ by application
   - ☐ by nomination
   - ☐ by invitation
   - ☐ other (please specify) ....
9. What are the eligibility criteria for becoming a member?
10. What is the role of the facilitator?
11. Is the CoP sponsored by an agency or organisation?

If so, as facilitator, what do you see as the role of the sponsoring agency?

12. What are the activities provided by the CoP?

**Activity**	**How often? (please indicate frequency in relevant column below)**								
	Daily	Weekly	Fortnightly	Monthly	Quarterly	Six-monthly	Annually	Less often	Never
Face-to-face meetings	☐	☐	☐	☐	☐	☐	☐	☐	☐
Face-to-face seminars	☐	☐	☐	☐	☐	☐	☐	☐	☐
Virtual meetings	☐	☐	☐	☐	☐	☐	☐	☐	☐
Virtual seminars (Webinars)	☐	☐	☐	☐	☐	☐	☐	☐	☐
Technical training courses delivered face-to-face	☐	☐	☐	☐	☐	☐	☐	☐	☐
Podcasted training sessions	☐	☐	☐	☐	☐	☐	☐	☐	☐
Teleconferences	☐	☐	☐	☐	☐	☐	☐	☐	☐
Blog	☐	☐	☐	☐	☐	☐	☐	☐	☐
Email ListServ	☐	☐	☐	☐	☐	☐	☐	☐	☐
Newsletter	☐	☐	☐	☐	☐	☐	☐	☐	☐
Social events	☐	☐	☐	☐	☐	☐	☐	☐	☐
Other (specify)	☐	☐	☐	☐	☐	☐	☐	☐	☐
Other (specify)	☐	☐	☐	☐	☐	☐	☐	☐	☐

13. Has the CoP ever been evaluated?

    Yes (go to Q14)

    No (skip to Q16)

14. Is it possible to get a copy of the report and data collection instruments please?

    Yes (go to Q16)

    No (go to Q15)

15. How was the impact of the CoPs assessed for the evaluation? *Multiple responses allowed*

- ☐ By surveying members
- ☐ By surveying stakeholders
- ☐ By collecting process indicator data (e.g. waiting times). Please specify indicators .....
- ☐ By collecting clinical indicator data (e.g. infection rates, readmission rats, etc.).

Please specify indicators used ....

- ☐ Other, please specify ....

16. Does the CoP report its activities and outcomes to any organisations? If so, what is the reason for this reporting?
17. Does the CoP collect data on a regular basis for reporting or other purposes?
    - ☐ Yes
    - ☐ No – skip to Q20
18. If yes, what are the indicators used in the data collection?
19. Is there any documentation that we can see demonstrating the impact of the CoP in improving performance against clinical indicators? For example, data collection instrument, report, etc.
20. To provide context, can you provide us with the following information?
    a. Number of professions represented in the membership
    b. Number of organisations represented in the membership
    c. The number of members located in regional and rural areas
    d. The number of members in metropolitan areas
21. In your opinion, how do you think CoPs facilitate the exchange of knowledge and best practice?
22. From a facilitator's perspective, how do you see the CoP benefiting the members as individuals?
23. Can you give two examples of such benefit to individual members?
24. How do you see the CoP benefiting the organisations for which the members work?

25. Can you give two examples of such benefit at an organisational level?
26. Do you see the CoP having any national significance? If so, how?
27. In your opinion, what has been the single most major achievement of this CoP?
28. From a facilitator's perspective, what are the main barriers to the CoP facilitating the process of improving practice in health care?
29. What are some factors that facilitate the role of the CoP in improving practice in healthcare?
30. Would you like to make any comments about the role of CoPs in improving organisational performance in healthcare?
