# Supplementary material for: A realist evaluation of the role of communities of practice in changing healthcare practice
Source: Implement Sci. 2011 May 23;6:49. doi: 10.1186/1748-5908-6-49 (PMC3120719; doi:10.1186/1748-5908-6-49)
Supplement: Additional file 2 — Survey of CoP members to test context, mechanism and outcome configurations. [file 1748-5908-6-49-S2.PDF]

Additional File 2: Survey of CoP members to test context, mechanism and outcome configurations

To be administered as an online survey

You are being asked to complete this online survey because you are a member of a community of practice that is participating in a research project conducted by the University of New South Wales. The purpose of this research is to understand how, why and when communities of practice improve clinical practice in the healthcare sector. This survey typically takes about 20 minutes to complete.

More details of the project are available in the information sheet that can be accessed through this link. Please do not hesitate to contact any member of the research team if you have any concerns or would like to discuss any aspect of the research.

Thank you for your time.

I. DEMOGRAPHICS

1. Your name .... (optional)

Your name will be replaced by a unique identifier during data entry. Data will be analysed at an aggregated level and no individual will be identifiable in the reporting of findings.

2. What is the name of the community of practice through which you were invited to participate? ....

II. HOW, WHY AND WHEN DO COMMUNITIES OF PRACTICE CHANGE WORK PRACTICE?

[Hypotheses to be tested will depend on the results of parts 1 and 2 of the realistic evaluation. The following hypotheses are listed as likely examples based on information gathered to date]

Please note that outside of the phrase 'communities of practice', the term 'practice' is used such that it refers to the carrying out of your work as it relates to your professional role.

In your opinion,

3. What did you hope to gain by participating in this CoP?
4. How much has being a member of this CoP impacted on your own work/practice? *Very much, somewhat, unsure,not much, not at all.*
5. How much has this CoP benefited the organisation that you work for? *Very much, somewhat, unsure, not much, not at all.*

The following set of your questions asks about the impact of your participation in the CoP on your work practice, as opposed to the impact at the organisational level.

Based on your experience with the CoP, how much do you agree with the following statements?

6. Participating in this CoP resulted in my adopting evidence-based guidelines/practice in my work practice. *Strongly agree ... agree ... unsure ... disagree ... strongly disagree*
7. Participating in this CoP resulted in my introducing a new method or approach to solving a problem that I experienced in relation to my work practice. *Strongly agree ... agree ... unsure... disagree ... strongly disagree*
8. Participating in this CoP resulted in my being able to complete a task or deliver an outcome in less than time that I would otherwise have required. *Strongly agree ... agree ... unsure ... disagree ... strongly disagree*
9. Participating in this CoP provided me with access to virtual networks, facilitating access to expertise not available locally. *Strongly agree ... agree ... unsure ... disagree ... strongly disagree*
10. Participating in this CoP provided me with the opportunity to discuss work-related problems in a non-judgemental environment. *Strongly agree ... agree ... unsure ... disagree ... strongly disagree*
11. Participating in this CoP provided me with access to experts in the field that I would otherwise have found difficult to obtain.

12. Getting access to multi-disciplinary relationships with other professionals through membership of the CoP helps me improve my work practice. *Strongly agree ... agree ... unsure ... disagree ... strongly disagree*
13. Participating in the CoP gives me access to information demonstrating successes of evidence-based practice *Strongly agree ... agree ... unsure ... disagree ... strongly disagree*
14. Getting access to information that demonstrates success of evidence-based practice (EBP) makes it more likely that I will adopt EBP in my practice. *Strongly agree ... agree ... undecided ... disagree ... strongly disagree*
15. Being a member of this CoP gives me access to a trusted colleague that I can turn to for advice or a second opinion, when needed. *Strongly agree ... agree ... undecided ... disagree ... strongly disagree*
16. Belonging to the same CoP helps establish trust with other members. . *Strongly agree ... agree ... unsure ... disagree ... strongly disagree*
17. Being a member of the CoP has helped me establish my professional identity. *Strongly agree ... agree ... undecided ... disagree ... strongly disagree*
18. The commitment of the other members of the CoP encourages my participation in the CoP. *Strongly agree ... agree ... undecided ... disagree ... strongly disagree*
19. Attending the seminars and other face-to-face meetings organised by the CoP helped me establish links with other clinicians involved in ED care. *Strongly agree ... agree ... undecided ... disagree ... strongly disagree*
20. Being a member of this CoP has impacted positively on my career potential. *Strongly agree ... agree ... undecided ... disagree ... strongly disagree*
21. Being a member of this CoP has helped at least on one occasion in my ability to solve a work-related problem. *Strongly agree ... agree ... undecided ... disagree ... strongly disagree*
22. Please list up to three definite examples of how your work/practice has changed as a result of your participation in activities and/or accessing resources provided by the CoP.
23. Which of the following best describes the frequency with which you read the CoP newsletter?
    - □ I usually read each issue in detail
    - □ I usually skim the contents of the newsletter and read only contents that I have a particular interest in
    - □ I usually leave it for reading later but more often than not, never get back to it
    - □ Rarely / never

24. How often do you participate in the following activities organised by the CoP?

	Every session	More often than not	Not often	Never
Face to face seminars				
Web seminars				
Face to face meeting of CoP members				
Teleconference of CoP members				
Blog discussions				
Email discussions via Listserve				
Other (Please specify)				
Other (Please specify)				
Other (Please specify)				

The following set of questions asks about the impact of this CoP on the organisation that you work for.

Based on your experience with the CoP, how much do you agree with the following statements?

25. The work of this CoP has resulted in my organisation successfully implementing evidence-based guidelines/practice. *Strongly agree ... agree ... unsure ... disagree ... strongly disagree*
26. The work of this CoP has resulted in my organisation developing a new systems or processes to improve services. *Strongly agree ... agree ... unsure ... disagree ... strongly disagree*
27. The work of this CoP has resulted in my organisation achieving improved clinical outcomes. *Strongly agree ... agree ... unsure ... disagree ... strongly disagree*
28. The work of this CoP has resulted in my organisation achieving improved patient satisfaction with services provided. *Strongly agree ... agree ... unsure ... disagree ... strongly disagree*
29. The work of this CoP has resulted in my organisation reducing time to solve at least one work related problem. *Strongly agree ... agree ... unsure ... disagree ... strongly disagree*

30. Please list up to three definite examples of how the work of this CoP has impacted on the delivery of services by the organisation that you work for? These examples may include an innovation, an introduction of a new procedure or process, and/or increased patient satisfaction with services provided by your organisation.

This is the end of the survey. Thank you for taking the time to complete the survey.
