# Supplementary material for: A realist evaluation of the role of communities of practice in changing healthcare practice
Source: Implement Sci. 2011 May 23;6:49. doi: 10.1186/1748-5908-6-49 (PMC3120719; doi:10.1186/1748-5908-6-49)
Supplement: Additional file 3 — Survey of CoP members to map the current structure, available expertise and knowledge exchange. [file 1748-5908-6-49-S3.PDF]

Additional File 3: Survey of CoP members to map the current structure, available expertise and knowledge exchange

To be administered as an online survey

You are being asked to complete this online survey because you are a member of a community of practice that is participating in a research project conducted by the University of New South Wales. This survey typically takes about 20 minutes to complete.

The information you provide by completing this questionnaire will only be seen by members of the research team. Unlike with the more common form of surveys, we are asking for your name to allow us to use social network analysis methods to examine professional and social connections and knowledge exchange that occur within the CoP. The names will be removed and replaced with an alphanumeric code at a professional level (e.g. Doctor 1, Nurse 1, etc.), when entering and analysing data. The unit of analysis is the community of practice and not the individual member; you will not be identifiable from the public reporting of the findings.

Social network analysis methods have been used in the healthcare sector to examine relationships among healthcare providers without compromising privacy, as demonstrated by the following examples:

- Creswick N, Westbrook JI, Braithwaite J. Understanding communication networks in the emergency department. *BMC Health Serv Res*. 2009 Dec 31;9:247.
- Creswick N, Westbrook JI. Social network analysis of medication advice-seeking interactions among staff in an Australian hospital. *Int J Med Inform*. 2010 Jun;79(6):e116-25. Epub 2008 Nov 12.
- Cott C. "We decide, you carry it out": A social network analysis of multidisciplinary longterm care teams. *Soc Sci Med.* 1997; 45(9), 1411-1421.
- Lurie SJ, Fogg TT, Dozier AM. (2009). Social network analysis as a method of assessing institutional culture: three case studies. *Acad Med*, 84(8), 1029-1035.

More details on the project are available in the information sheet that can be accessed through this link. Please do not hesitate to contact any member of the research team if you have any concerns or would like to discuss any aspect of the research.

Thank you for your time.

I. DEMOGRAPHICS

1. Your name ....

Your name will be replaced by a unique identifier during data entry. Data will be analysed at an aggregated level and no individual will be identifiable in the reporting of findings.

2. What is the name of the community of practice through which you were invited to participate? ....

3. What is your sex? Male
   Female

4. What age group do you belong to? <20 years
   20 – 34 years
   35 – 49 years
   50 – 69 years
   70 + years

5. What is the name of your primary workplace/practice? ....

6. In which State or Territory is this workplace/practice located?

7. In which city or town is this workplace/practice located?

8. What is the position that you currently hold at your primary workplace/practice?

   Nursing Unit Manager
   Clinical Nurse Consultant
   Clinical Nurse Specialist
   Registered Nurse
   Enrolled Nurse
   Nurse educator
   Student Nurse
   Senior Allied Health staff
   Member of the allied health team
   Resident Medical Officer
   Registrar
   Staff specialist
   VMO
   Hospital scientist
   Academic
   Executive / Senior Management /Management position
   Administrative/project staff
   Other – please specify ....

10. How many years have you been working in this profession?

   .... years
   OR
   .... months if less than a year

11. How long have you been a member of this CoP?
   .... years
   OR
   .... months if less than a year

12. Are you considered an expert in an area relevant to the CoP?
   Yes/No (If no, skip Q14)

13. If yes, what is your area of expertise? ....

14. Which of the following best describes the frequency with which you read the CoP newsletter?

- □ I usually read each issue in detail
- □ I usually skim the contents of the newsletter and read only contents that I have a particular interest in
- □ I usually leave it for reading later but more often than not, never get back to it
- □ Rarely / never

15. How often do you participate in the following activities organised by the CoP?

	Every session	More often than not	Not often	Never
Face to face seminars				
Web seminars				
Face to face meeting of CoP members				
Teleconference of CoP members				
Blog discussions				
Email discussions via Listserve				
Other (Please specify)				
Other (Please specify)				
Other (Please specify)				

II. INFORMATION AND KNOWLEDGE EXCHANGE WITHIN THE COP

The next set of questions asks about exchange of information and knowledge to and between members of the CoP and about interactions that you have with other members of the CoP. The names are provided to help identify interactions and will be removed for data analysis and reporting. The analysis will be done at a CoP level and not an individual level. No person will be identifiable from the reporting of the results.

Please use the table that accompanies each question when responding to each question. When filling in this table, please:

- *Provide a response against all names in the table.*
- *Select the 'self' option against your own name.*

This table will follow each question

	Self	Rarely/Never	Yearly	Monthly	Weekly	Daily
Name 1						
Name 2						
Name 3						
...						
...						
...						
...						

Note: The time frame of a year was chosen to allow capture of activities such as face-to-face seminars that may not be held frequently.

16. In the last year, on average, how often have you met face-to-face with the following people?
17. In the last year, on average, how often have you communicated via telephone or email with the following people? We are interested in virtual communication initiated by either party.
18. In the last year, on average, how often have you socialised (e.g. had lunch or coffee) with the following people? (table)
19. In the last year, on average, how often have you asked this person for help or advice on a work-related matter? (table)
20. In the last year, on average, how often have you received information from the following people that has changed or influenced your work practice? (table)
21. How does interacting with each person below typically affect your energy levels? (table with not at all, not much, uncertain, somewhat, very much options against each name).
22. Did you know this person socially and/or professionally prior to joining this CoP? (Table with yes / no options against each name)
23. In your opinion, what can the CoP do to help improve the work that you and your organisation provide in delivering healthcare?
24. Are there any comments you would like to make in relation to assessing the value of the CoP in improving healthcare practice?

This is the end of the survey. Thank you for taking the time to complete the survey.
